# Low-intensity pulsed ultrasound upregulates osteogenesis under inflammatory conditions in periodontal ligament stem cells through unfolded protein response

**DOI:** 10.1186/s13287-020-01732-5

**Published:** 2020-06-03

**Authors:** Han Li, Yuejia Deng, Minmin Tan, Ge Feng, Yunchun Kuang, Jie Li, Jinlin Song

**Affiliations:** 1 College of Stomatology, Chongqing Medical University, Chongqing, China; 2 Chongqing Key Laboratory of Oral Diseases and Biomedical Sciences, Chongqing, China; 3 Chongqing Municipal Key Laboratory of Oral Biomedical Engineering of Higher Education, Chongqing, China; 4 Department of Biomedical Sciences, College of Dentistry, Texas A&M University, Dallas, TX 75246 USA

**Keywords:** Periodontitis, PDLSCs, Endoplasmic reticulum stress, LIPUS, Inflammation, Osteogenic differentiation

## Abstract

**Background:**

In periodontal tissue engineering, periodontal ligament stem cells derived from patients with periodontitis (P-PDLSCs) are among the most promising and accessible stem cells for repairing disrupted alveolar bone and other connective tissues around the teeth. However, the inflammatory environment influences the osteogenic differentiation ability of P-PDLSCs. We examined low-intensity pulsed ultrasound (LIPUS) in P-PDLSCs in vitro and in rats with experimental periodontitis to determine whether LIPUS can enhance the osteogenic differentiation of stem cells.

**Materials and methods:**

P-PDLSCs were harvested and isolated from the periodontal tissues around the teeth of periodontitis patients, and healthy PDLSCs (H-PDLSCs) were obtained from tissues around healthy teeth. After validation by flow cytometry analysis, the P-PDLSCs were cultured in osteogenic medium either pretreated with the endoplasmic reticulum stress (ERS) inhibitor 4-phenyl butyric acid (4-PBA) or not pretreated and then treated with or without LIPUS (90 mW/cm^2^, 1.5 MHz) for 30 min per day. Cell viability, ERS marker expression, and osteogenic potential were determined between the different treatment groups. LPS-induced H-PDLSCs were used to mimic the inflammatory environment. In addition, we established a model of experimental periodontitis in rats and used LIPUS and 4-PBA as treatment methods. Then, the maxillary bone was collected, and micro-CT and histology staining methods were used to detect the absorption of alveolar bone.

**Results:**

Our data showed that the P-PDLSCs derived from periodontitis tissues were in a more pronounced ERS state than were the H-PDLSCs, which resulted in the former being associated with increased inflammation and decreased osteogenic ability. LIPUS can alleviate ERS and inflammation while increasing the bone formation capacity of P-PDLSCs in vivo and in vitro.

**Conclusions:**

LIPUS may be an effective method to enhance the outcome of periodontal tissue engineering treatments of periodontitis by suppressing inflammation and increasing the osteogenic differentiation of P-PDLSCs through the unfolded protein response pathway, and more detailed studies are needed in the future.

## Background

Periodontitis is a chronic inflammatory disease affecting approximately 90% of the population worldwide. Periodontal pathogenic bacteria such as *Porphyromonas gingivalis* (*P*. *gingivalis*) may initially contribute to the development of immune processes in immune and resident cells in tissue with chronic periodontitis [1]. The chronic inflammation of periodontitis mainly destroys the alveolar bone and the connective tissue that supports teeth [2], which can induce gradual tooth detachment from the periodontal ligament (PDL), periodontal pocket formation, and ultimately tooth loss.

In the condition of periodontitis, the bone repair and reconstruction is impaired. Conventional clinical treatment cannot completely restore damaged periodontal tissue. In clinical practice, the standard mechanical method is commonly used to remove subgingival biofilms and calculus, which can reduce clinical attachment loss in a short period [3]. However, infrequent maintenance leads to chronic periodontitis, which recurs even with the application of antibacterial adjuvants [4]. The repair of periodontium tissue remains an ongoing challenge.

In recent years, cell tissue engineering (CTE) technology has become a promising tissue repair strategy [5], in which stem cells are one of the three basic elements [6]. In periodontal tissue engineering, dental cells present advantages. Human periodontal ligament stem cells (PDLSCs) are a group of mesenchymal stem cells (MSCs) derived from the periodontal ligament, which have tissue specificity and multidirectional differentiation capability, endowing them with potential application value in periodontal tissue regeneration [7, 8]. Currently, most PDLSCs are derived from premolars extracted for orthodontic purposes, but the number of PDLSCs derived from healthy teeth (H-PDLSCs) is limited. Liu et al. reported that PDLSCs derived from periodontitis-affected teeth (P-PDLSCs) also have the characteristics of stem cells [9], which may have a positive effect on periodontal tissue regeneration because P-PDLSCs are obtained from a periodontitis patient seeking treatment, reducing the need for cells derived from other individuals and preventing immune rejection.

However, the inflammatory state of the cells inhibits their osteogenic differentiation potential [10], impeding the repair and reconstruction of periodontal tissues. Xue et al. reported that P-PDLSCs are in an endoplasmic reticulum stress (ERS) state [11], which is a pathological condition that induced the unfolded protein reaction (UPR) in cells to defend against the threat of ER Ca^2+^ depletion, metabolic stimuli, defective glycosylation, energy deprivation, altered redox status, increased protein secretion, and inflammatory stimuli [12]. Hisanori et al. reported a significant increase in the expression of UPR-related genes in periodontitis [13]. The prolonged inflammation of chronic periodontitis triggers UPR, leading to decreased osteogenic differentiation, whereas Xue et al. reported that reducing ERS in a rat alveolar bone defect model increased the osteogenic capacity of P-PDLSCs [11]. In addition, Yamada et al. found that ERS stimulated by *P. gingivalis* in experimental periodontitis in mice was involved in the alveolar bone resorption process [14]. Recently, Yang et al. illustrated that osteoblast differentiation of PDLCs under cyclic mechanical forces was enhanced by UPR and found that PERK−/− PDLCs cells showed decreased osteogenic differentiation [15]. These contradictory results from studies on the relationship between ERS and osteogenic differentiation have yet to be explored and require further research, especially or those findings in which mechanical forces are involved.

In our previous study, we use low-intensity pulsed ultrasound (LIPUS) as a non-invasive mechanical stimulation that facilitated osteogenesis of PDLSCs [16] and alleviated the expression of lipopolysaccharide (LPS)-induced inflammatory factors [17]. LIPUS is thought to be an effective potential method for the treatment of periodontitis in the future. Recently, Su et al. reported that LIPUS activated ERS through the p38-mapk-mediated signaling pathway, which promoted the apoptosis of malignant solid tumor cells and inhibited angiogenesis induced by human umbilical vein endothelial cells, showing that LIPUS is an alternative to inhibit tumor growth [18]. However, the functional mechanism of LIPUS is unclear, especially when applied to PDLSCs. To explore the mechanism, we used LIPUS to treat P-PDLSCs and LPS-induced H-PDLSCs and then observed the effects on cells on the basis of changes in proliferation and differentiation ability, including changes in ERS-associated marker expressions, inflammation level, and osteogenesis in vitro. Additionally, we established an experimental periodontitis model in rats through ligation and LPS injection. LIPUS was used to treat these animal models to observe the progression of periodontitis in different groups. The object of our study was to examine the effects of LIPUS on osteogenic differentiation and inflammation induction of PDLSCs in inflammatory states and to determine whether the UPR plays a role in this process.

## Materials and methods

### Isolation and culture of primary cells (H-PDLSCs and P-PDLSCs)

The protocol was approved by the Ethics Committee of the Affiliated Stomatological Hospital of Chongqing Medical University. Patients (age 16 and above 16) and parents/legally authorized representatives of healthy participants (age below 16) have agreed and signed the informed consent documents before the study started. H-PDLSCs were extracted from healthy teeth of 10 people (12–25 years old) with orthodontic reasons. P-PDLSCs were obtained from the teeth of 15 patients (25–40 years old) who were clinically diagnosed with periodontitis.

Briefly, the periodontal membrane tissue was scraped off with a blade in the middle third of the root. Tissue fragments were then digested with 3 mg/ml type Ι collagenase (Sigma, USA) in a 37 °C water bath for 30 min. Finally, the cells were cultured in α-minimum essential medium (α-MEM) (HyClone, USA) containing 10% fetal bovine serum (FBS, Gemini, USA) and 1% streptomycin and penicillin (HyClone, USA) at 37 °C with 5% CO_2_. Replace the medium every 3 days and all experiments were performed with cells from passages 3 to 4 (P3-P4). For osteogenic induction, H-PDLSCs and P-PDLSCs were seeded in six-well culture dishes at a density of 1 × 10^4^ cells/cm^2^. Upon reaching 70% confluence, cells were cultured in the osteogenic medium (OM) supplemented with 5 mM β-glycerophosphate (Sigma, USA), 50 μg/ml ascorbic acid (Sigma, USA), and 10 nM dexamethasone.

### Flow cytometry analysis

For immunophenotype characterization, H-PDLSCs and P-PDLSCs were trypsinized, resuspended, and incubated with anti-human stem cell surface-labeled antibodies including human CD34-phycoerythrin (PE) (1:100, BD Biosciences, USA), CD73-PE (1:100, BD Biosciences), and CD146-PE (1:100, BD Biosciences) for 1 h at 4 °C. Cells not treated with fluorescent antibodies were used as a control group. Flow cytometry was performed on a BD Accuri™ C6 flow cytometer (BD Biosciences).

### Alkaline phosphatase (ALP) staining

H-PDLSCs and P-PDLSCs were seeded in 6-well plates at the same initial density. After 7 days of culture in OM, the cells were washed 3 times with PBS, fixed with 4% paraformaldehyde (Sigma Aldrich, USA) for 15 min, and then washed again with PBS. Staining was performed using an alkaline phosphatase staining kit (Beyotime, China) according to the manufacturer’s instructions. Finally, the cells were gently washed with double distilled water, and images were taken with a camera and an inverted microscope (Primo Vert monitor).

### Alizarin red staining

Cells of the same density were cultured in a 6-well plate as described above, and the formation of calcium nodules was observed after 21 days of culture in OM. The cells were gently washed with PBS, fixed with 4% paraformaldehyde for 15 min, and stained with a 0.02% Alizarin Red Staining Kit (Solarbio, China) for about 20 min at room temperature. Finally, the excess dye was removed by gentle washing with double distilled water. The images were obtained with a camera and an inverted microscope. For quantification analysis, 10% hexadecyl pyridinium chloride monohydrate (CPC) was used to dissolve the mineralized nodules and then measured the colorimetric absorbance at 562 nm.

### Cell proliferation assay

Cell proliferation of PDLSCs in different treatment groups was tested using Cell Counting Kit-8 (CCK-8; Dojindo, Japan). The cells were seeded in 96-well plates at an initial density of 2 × 10^4^ cells/ml. After the cells were attached, 100 μl of medium containing 10 μl of CCK-8 was added, followed by incubation at 37 °C for 3 h, and the absorbance was measured at 450 nm by a spectrophotometer (Thermo Fisher, USA). Four parallel replicates were prepared.

### Transmission electron microscopy (TEM)

The cells were washed and digested by 0.25% trypsin for 1 min, harvested and centrifuged (3000*g*) for 10 min in a high-speed centrifuge, then fixed with 4% glutaraldehyde and 4% paraformaldehyde (diluted in phosphate-buffered saline, pH 7.2), and dehydrated and embedded for sectioning. Ultrathin sections were stained with uranyl acetate (30 min) and lead citrate (10 min) and observed by Hitachi-7500 transmission electron microscope (Hitachi Company, Japan) with an accelerating voltage of 100 kV.

### Quantitative real-time PCR analysis (qRT-PCR)

Briefly, total RNA was obtained using RNAiso Plus (TaKaRa, Dalian, China) according to the manufacturer’s standard protocol, and then transcribed in a 10-μl reaction volume using the Prime Script™ RT Reagent Kit (TaKaRa, Dalian, China). The cDNA was amplified using the SYBR-Premix Ex Taq™ kit (TaKaRa) for qRT-PCR and detected on a CFX96 Touch™ Real-time system (Bio-Rad, USA). The process was as follows: initial degeneration at 95 °C for 30 s; 40 cycles of amplification at 95 °C for 5 s, 60 °C for 30 s; and then 45 PCR cycles at 95 °C for 10 s, 65 °C for 5 s. Gene expression levels were calculated using the 2^-ΔΔCT^ method normalized to the reference gene GAPDH. Table 1 shows all primer sequences used in this study.

**Table 1:** Primer sequence information

Gene	Forward primer sequence (5′-3′)	Reverse primer sequence (5′-3′)
IL-6	AAGGTGGGTGTGTCCTCATTC	TTGCATCCCTGAGTTGTCCAG
IL-8	TGGACCCCAAGGAAAACTGG	GCTTGAAGTTTCACTGGCATCT
GRP78	TGCCCATCTCTGGAAGCCTA	CCGCAGTCAAGATGCCAAAC
IRE1α	AGCCGTGGTGAAGATGGACT	TCTCATGGCTCGGAGGAGAT
ATF4	CGTCCTCGGCCTTCACAATAA	CCTCACGAAAGGAGAGAGGTG
CHOP	TTCTCTGGCTTGGCTGACTG	TCCTCCTCTTCCTCCTGAGC
XBP1u	GTTAAGACAGCGCTTGGGGA	TGCACGTAGTCTGAGTGCTG
XBP1s	CTGAGTCCGCAGCAGGTG	GTCCAGAATGCCCAACAGGA
GAPDH	AGCCGCATCTTCTTTTGCGTC	TCATATTTGGCAGGTTTTTCT

### Western blot

Total protein was harvested using a RIPA Kit (Beyotime, China) with the addition of 1 mM protease inhibitor PMSF at 4 °C. After centrifugation for 10 min at 4 °C (12,000 RPM), the protein concentration was determined using the bicinchoninic acid protein assay kit (Beyotime, China) according to the instructions. The protein was loaded onto a 10% sodium dodecyl sulfate-polyacrylamide gel electrophoresis and then transferred to a polyvinylidene fluoride membrane (Millipore, USA). The membrane was blocked with TBST (TBS containing 0.1% Tween) containing 5% skim milk powder for 1–2 h and then incubated with primary antibodies including anti-IRE1α (1:1000, Abcam), anti-pIRE1α(1:1000, Abcam), anti-runt-related transcription factor 2 (Runx2; 1:1000, CST), anti-PERK (1:1000, Abcam), anti-GRP78 (1:1000, Abcam), anti-PDI (1:1000, Abcam), and glyceraldehyde 3-phosphate dehydrogenase (GAPDH; 1:1000, Zen-bio, China) at 4 °C overnight. The next day, the membrane was incubated with anti-goat or anti-mouse IgG secondary antibodies (Bio-rad, USA) conjugated with horseradish peroxidase (HRP) at room temperature for 2 h before being visualized by hypersensitive ECL chemiluminescence kit (Beyotime, China). Quantification of Western blot data was performed using ImageJ software.

### Enzyme-linked immunosorbent assay (ELISA)

Similarly, H-PDLSCs and P-PDLSCs were seeded at the same initial density into 6-well plates. Upon the cells were grown to 40%, they were pretreated with 4-PBA for 8 h, followed by LPS treatment for 96 h, and then subjected to LIPUS for 30 min, and finally, the supernatant was collected for the study. According to the manufacturer’s instructions, the protein levels of IL-6 and IL-8 in the supernatant were determined by an ELISA kit (BOSTER, China).

### In vivo experimental design

Animals used in this study were maintained in accordance with the Guideline for ethical review of animal welfare of Laboratory animals published by the China National Standardization Management Committee (publication No. GB/T 35892-2018) and were approved by the Ethics Committee of the Stomatological Hospital Affiliated to Chongqing Medical University (CQMU). Twenty healthy male Sprague-Dawley (SD) rats (8 weeks old) were obtained from the CQMU Animal Experimental Center. In this study, all rats weighed approximately 200–250 g. Rats were fed standard food regularly without any disease.

Twenty SD rats were randomly divided into 4 groups (*n* = 5 per group): the control group (no periodontitis, G1), the experimental periodontitis group (ligature + LPS, G2), the LIPUS treatment group (ligature + LPS + LIPUS, G3), and the 4-PBA treatment group (ligature + LPS + 4-PBA, G4). General anesthesia was injected intraperitoneally with 10% chloral hydrate (5 mL/kg) (Aladdin, China), and surgery was performed by two investigators. The gingiva of the maxillary first molar was separated with a dental probe, and then the 0.020 stainless steel ligature was inserted into the distal side and ring the maxillary first molar, and the ligature needs to be tied tightly. 0.1 ml LPS (10μg/ml) was injected at 48-h intervals, 4 times in total. After induction of periodontitis, G3 received treatment of LIPUS (90 mW/cm^2^,1.5 mHz,30 min) for 28 days, and G4 was fed with 4-PBA solution at a final concentration of 5 mg/kg body weight through drinking water for 28 days. Eight weeks after the induction of periodontitis, the rats (*n* = 5 per group) were euthanized and samples were obtained for further experimental analyses.

### Micro-computed tomography and bone morphometric analysis of rats

Eight weeks after the induction of periodontitis, maxillary jaws were scanned using a micro-CT system (BRUKER skyscan1176) with software including NRecon reconstruction, CTAn 1.8 and CTvox. In this study, the X-ray source was set to 70 kV and 357 μA, the 1-mm filter had a pixel size of 17.7 μm, and the X-ray tomography had a rotation angle of 180° (rotation step of 0.5°). The vertical distance from the lowest point of the crown enamel to the non-discrete bone height of the target root (ABLs, μm) and the vertical distance from the lowest point of the crown enamel of the section of the root canal to the lowest point of the target root apical (RBLs, μm) were measured for the calculation of the absorption of the alveolar bone ratio (ABLs/RBLs).

### Hematoxylin-eosin (H&E) staining

The obtained samples were fixed with 4% paraformaldehyde overnight, decalcified in 10% ethylenediaminetetraacetic acid (EDTA) (pH = 8.0) at 37 °C for 2 months, and changed medium every 2 days. Dehydrate with a series of ethanol and embed in paraffin. Then, 5-μm-thick paraffin sections were prepared according to the instructions for H&E staining to evaluate periodontal regeneration.

### Statistical analysis

All experiments were repeated at least three times to confirm the reliability of the study. Data were submitted as mean ± standard deviation (SD). Statistical significance was analyzed by SPSS (version 13.0; SPSS, Chicago, IL). It was considered statistically significant when *p* < 0.05.

## Results

### Morphology and characteristics of P-PDLSCs from the teeth of patients with chronic periodontitis and of H-PDLSCs from healthy teeth

**Fig. 1:**
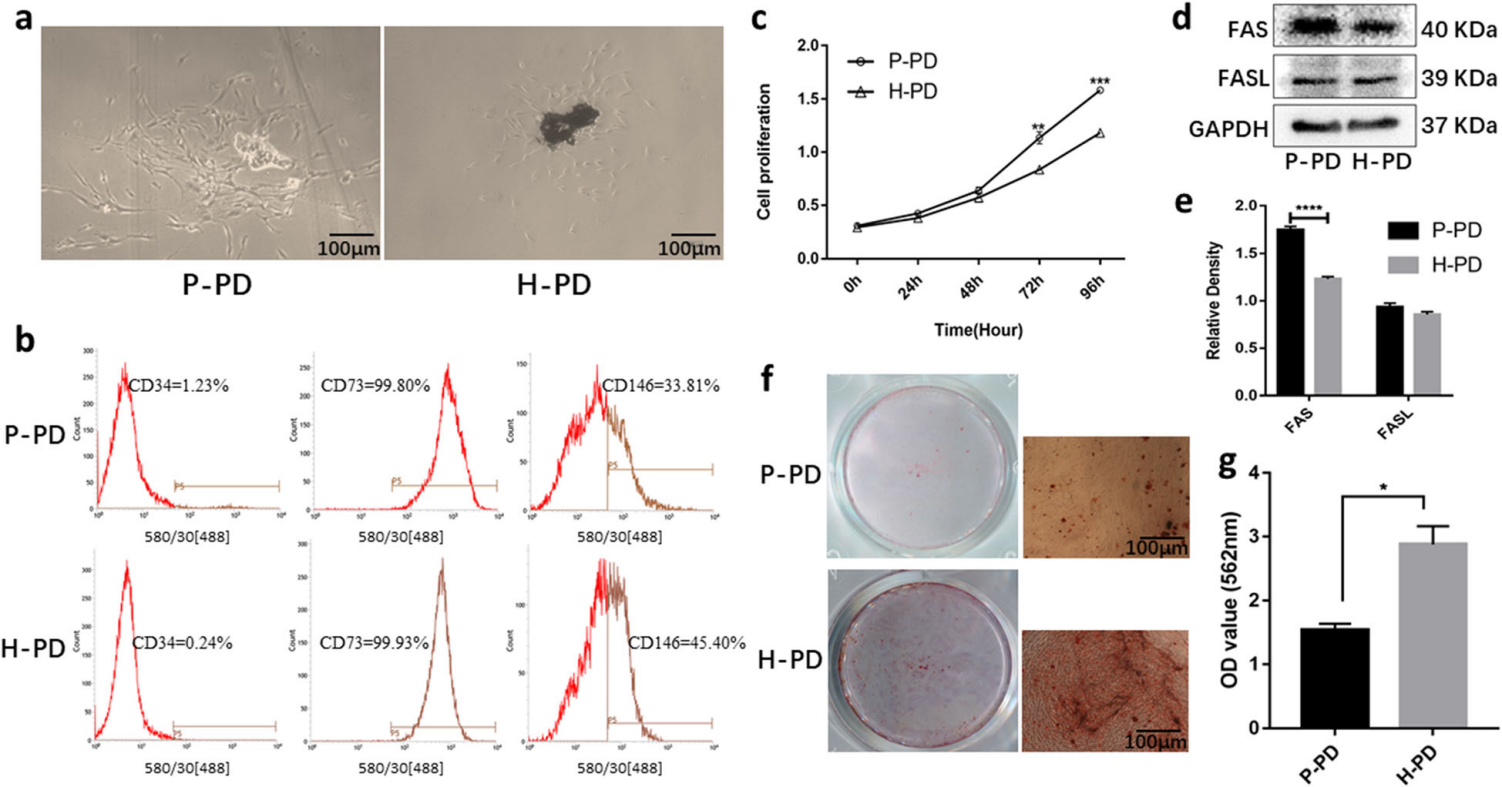
Comparison of P-PDLSCs and H-PDLSCs. **a** Primary cells grown up around the tissue mass. **b** Flow cytometry analysis of P-PD and H-PD. Cells were cultured in P3 or P4 and the results showed that P-PD and H-PD were positive for CD73 and CD146, but negative for CD34. **c** The proliferation of inflamed and normal PDLSCs were determined by CCK-8 on days 0, 1, 2, 3, and 4 (*n* = 5). There was a significant difference in the rate of proliferation after 3 days. **d**, **e** Western blot was used to detect apoptosis-related FAS/FASL proteins (**d**), and the corresponding relative expression levels were measured by ImageJ software (**e**) (*n* = 3). **f** After osteogenic induction for 21 days, cells were stained with Alizarin Red S to detect matrix mineralization. P-PD had less mineralized calcium nodules comparing with H-PD. **g** The result of calcium nodules quantification assay (*n* = 3). The data are expressed as means ± SD (*p < 0 .05; **p < 0 .01; ***p < 0 .001; ****p < 0 .0001, compared to the H-PD group). P-PD, P-PDLSCs; H-PD, H-PDLSCs

Primary P-PDLSCs and H-PDLSCs in the shape of fibroblast spindles were observed around the tissue pieces, and P-PDLSCs presented with a longer cell morphology (Fig. 1a). The flow cytometry results showed that both P-PDLSCs and H-PDLSCs exhibited positive mesenchymal stem cell surface markers, including CD73 and CD146, whereas the hematopoietic marker CD34 was negative (Fig. 1b). The viability of the two cells was tested by CCK-8 assay, and the results displayed that the proliferation rate of the P-PDLSCs was significantly higher than that of the H-PDLSCs after 3 days (*p* < 0.01) (Fig. 1c). Western blot was used to detect apoptosis-related FAS/FASL proteins. FAS expression in P-PDLSCs was significantly increased (Fig. 1d, e). Alizarin red staining was performed 21 days after osteogenic induction to observe the deposition of calcium. We found that the P-PDLSCs showed fewer mineralized calcium nodules than did the H-PDLSCs (*p* < 0.05) (Fig. 1f, g), suggesting a decrease in osteogenic capacity of the P-PDLSCs.

### Increased inflammatory factors and impaired osteogenesis in P-PDLSCs may be related to the UPR activating by chronic inflammation

**Fig. 2:**
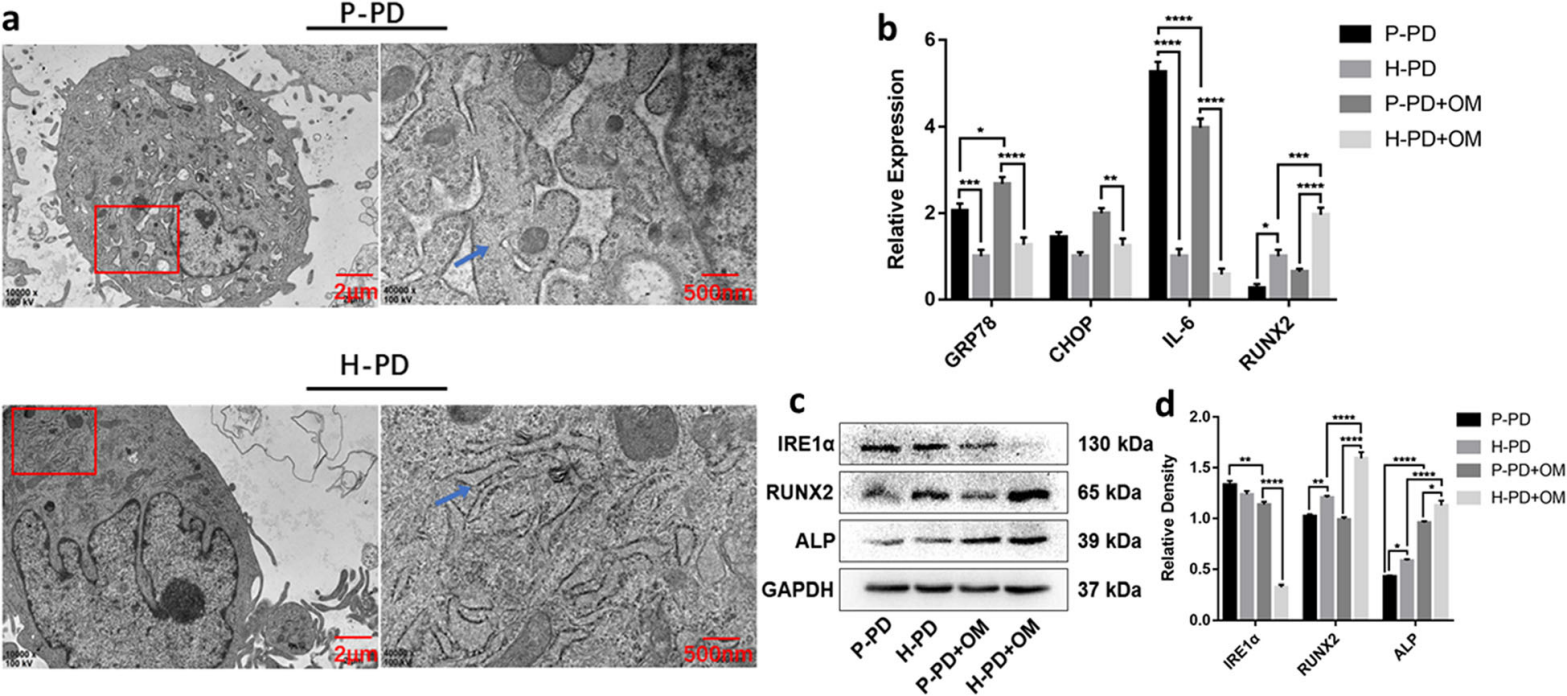
The activation of UPR in P-PD. **a** Transmission electron microscopy (TEM) images of the endoplasmic reticulum (ER) in P-PD and H-PD. The red box is the area of interest, and the blue arrow indicates the location of ER. **b** qRT-PCR was performed to detect the expression of UPR-related gene GRP78 and CHOP, inflammatory factor IL-6 and osteogenic differentiation markers RUNX2 in P-PD, H-PD, P-PD + OM, and H-PD + OM. **c**, **d** Western blot detected the UPR target protein IRE1α and the osteo-related proteins RUNX2 and ALP (**c**), and the corresponding relative expression levels were measured by ImageJ software (**d**). All data are presented as means ± SD (*n* = 3). The representative results were from three independent experiments (*p < 0 .05; **p < 0 .01; ***p < 0 .001; ****p < 0 .0001). P-PD, P-PDLSCs; H-PD, H-PDLSCs; P-PD + OM, P-PDLSCs + osteogenic medium; H-PD + OM, H-PDLSCs + osteogenic medium

We observed morphological changes in the ER using transmission electron microscopy (TEM) and found some swelled cisternae and degranulation in the P-PDLSCs that were not observed in the H-PDLSCs (Fig. 2a), indicating that the chronic inflammatory environment may induce ERS. After 7 days of osteogenesis induction, the expression of ERS, inflammation, and osteo-associated markers were compared with undifferentiated P-PDLSCs and H-PDLSCs by qRT-PCR and western blot. qRT-PCR found that the expression of UPR-related molecular chaperone glucose-regulated protein 78 (GRP78) in undifferentiated P-PDLSCs was higher than in H-PDLSCs, and CHOP had a tendency to increase but was not statistically significant. After osteogenesis induction, the expressions of GRP78 and CHOP increased and were significantly higher in the P-PDLSCs than in the H-PDLSCs (Fig. 2b). Whether induced differentiation or not, compared with H-PDLSCs, the expression of the inflammatory cytokine IL-6 was significantly increased in P-PDLSCs and the osteogenic marker RUNX2 was decreased. After osteogenesis induction, IL-6 expression decreased and RUNX2 increased further (Fig. 2b). Western blot analysis revealed that the expression of osteo-associated proteins RUNX2 and ALP in P-PDLSCs were significantly lower than in H-PDLSCs with or without osteogenesis induction (Fig. 2c, d). And the transmembrane protein IRE1α, which is involved in the regulation of ERS, was significantly higher in P-PDLSCs than in H-PDLSCs under osteogenic conditions (Fig. 2c, d). These findings indicated that periodontitis-derived P-PDLSCs highly expressed inflammatory factors and significantly impaired osteogenesis, which may be related to the activation of UPR.

### LIPUS inhibits inflammation and enhances osteogenesis ability of P-PDLSCs by reducing UPR

**Fig. 3:**
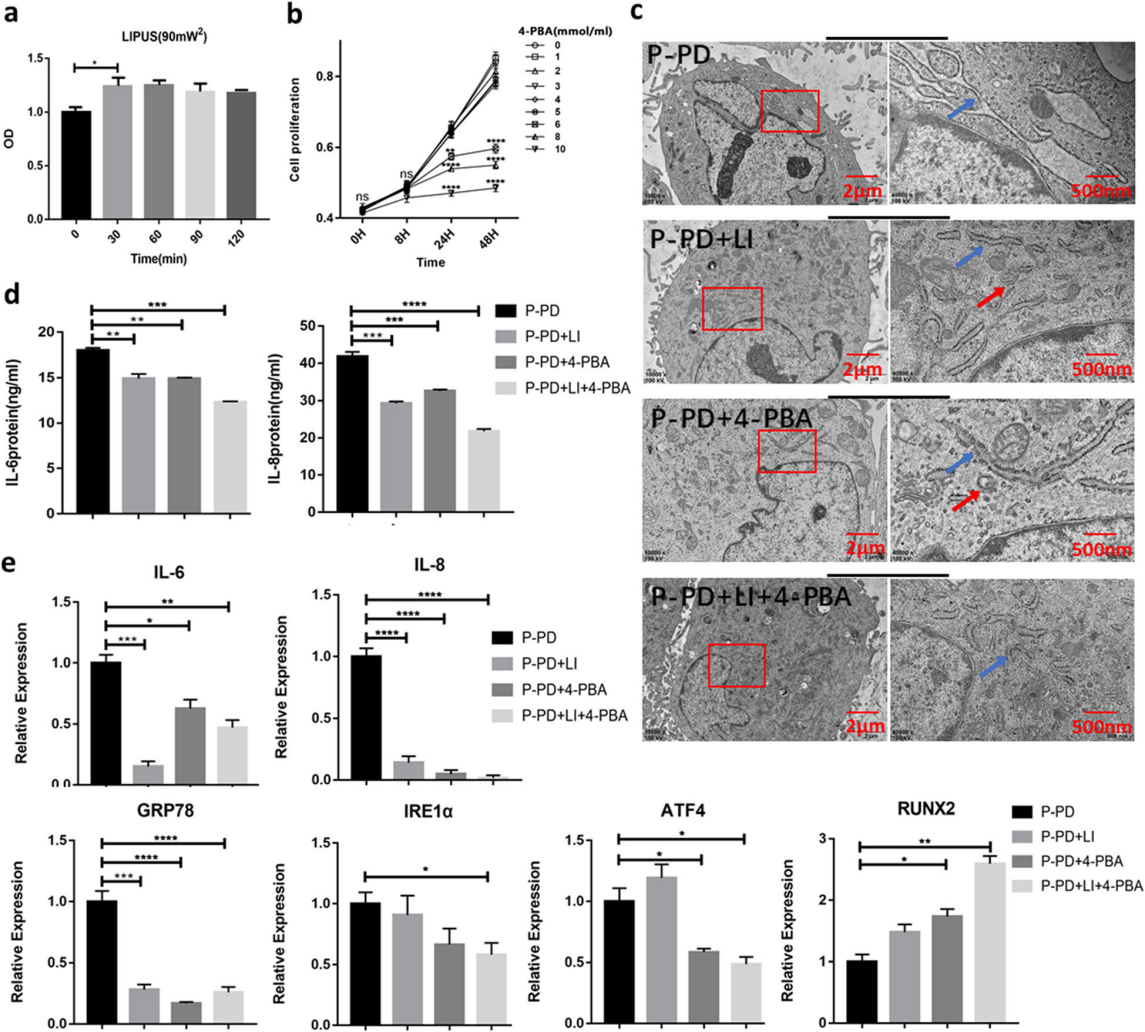
LIPUS may reduce the expression of inflammatory factors in P-PDLSCs by inhibiting ERS. **a**, **b** CCK-8 assay optimized the intensity of LIPUS (**a**) and the dose of 4-PBA (**b**) (*n* = 5). **c** TEM images observed the morphology of ER. The red box is the area of interest, the blue arrow indicates the location of ER, and the red arrow refers to the autophagosome. **d** ELISA was used to detect the secretion of IL-6 and IL-8 inflammatory factors (*n* = 3). **e** LIPUS and 4-PBA modulated the levels of IL-6, IL-8, GRP78, IRE1α, ATF4, and Runx2 by qRT-PCR analysis (*n* = 3). Gene expression was normalized to GAPDH. All data are presented as means ± SD. The representative results were from three independent experiments (ns, no significant; *p < 0 .05; **p < 0 .01; ***p < 0 .001; ****p < 0 .0001). P-PD, P-PDLSCs; H-PD, H-PDLSCs; LI, LIPUS; H, hour

The small molecule compound 4-phenylbutyric acid (4-PBA) can enhance the folding capacity of the endoplasmic reticulum, stabilize protein conformation, and promote the transport of mutant proteins, thereby inhibiting ERS [19]. To optimize the intensity of LIPUS and the dose of 4-PBA, the effects of LIPUS after different treatment times (30 min, 60 min, 90 min, 120 min) and at different concentrations of 4-PBA (from 1 to 10 mM) (Solarbio, China) treatment on the proliferation of PDLSCs were determined by CCK-8 assay. Our results demonstrated that LIPUS irradiation for 30 min significantly promoted cell proliferation (Fig. 3a) and that 4-PBA (5 mM) pretreatment for 8 h had little effect on cell proliferation and induced low levels of cytotoxicity (Fig. 3b). Therefore, in the experiments, we irradiated cells with LIPUS (90 mW/cm^2^, 1.5 MHz) for 30 min and pretreatment with 4-PBA (5 mM) for 8 h.

The P-PDLSCs were divided into four groups: (1) P-PDLSCs, (2) P-PDLSCs + LIPUS, (3) P-PDLSCs + 4-PBA, and (4) P-PDLSCs + LIPUS + 4-PBA. The LIPUS group was continuously irradiated with LIPUS for 7 days, and the 4-PBA group was pretreated with 4-PBA for 8 h. The fourth group was first pretreated with 4-PBA, followed by LIPUS irradiation for 30 min/day for 1 week. First, by TEM, we observed the morphology of the ER in different groups by TEM analysis. The results showed that compared with the ER in the P-PDLSCs group, regardless of whether LIPUS and 4-PBA were used individually or in combination, the extent of cisterna dilation was decreased in the ER, which returned to normal size and shape (Fig. 3c). Then, the expression of the inflammatory secretory proteins IL-6 and IL-8 was measured by ELISA. We found that both LIPUS and 4-PBA decreased the expression of IL-6 and IL-8 in P-PDLSCs, and this reduction was most obvious when both treatments were used (Fig. 3d). The results from qRT-PCR also indicated a significant decrease in IL-6 and IL-8 after LIPUS and 4-PBA treatment, and the UPR-related gene GRP78, inositol requiring enzyme1α (IRE1α), and activating transcription factor (ATF4) were also downregulated. Nevertheless, the osteogenic marker gene RUNX2 level was dramatically increased after the above treatment (Fig. 3e).

**Fig. 4:**
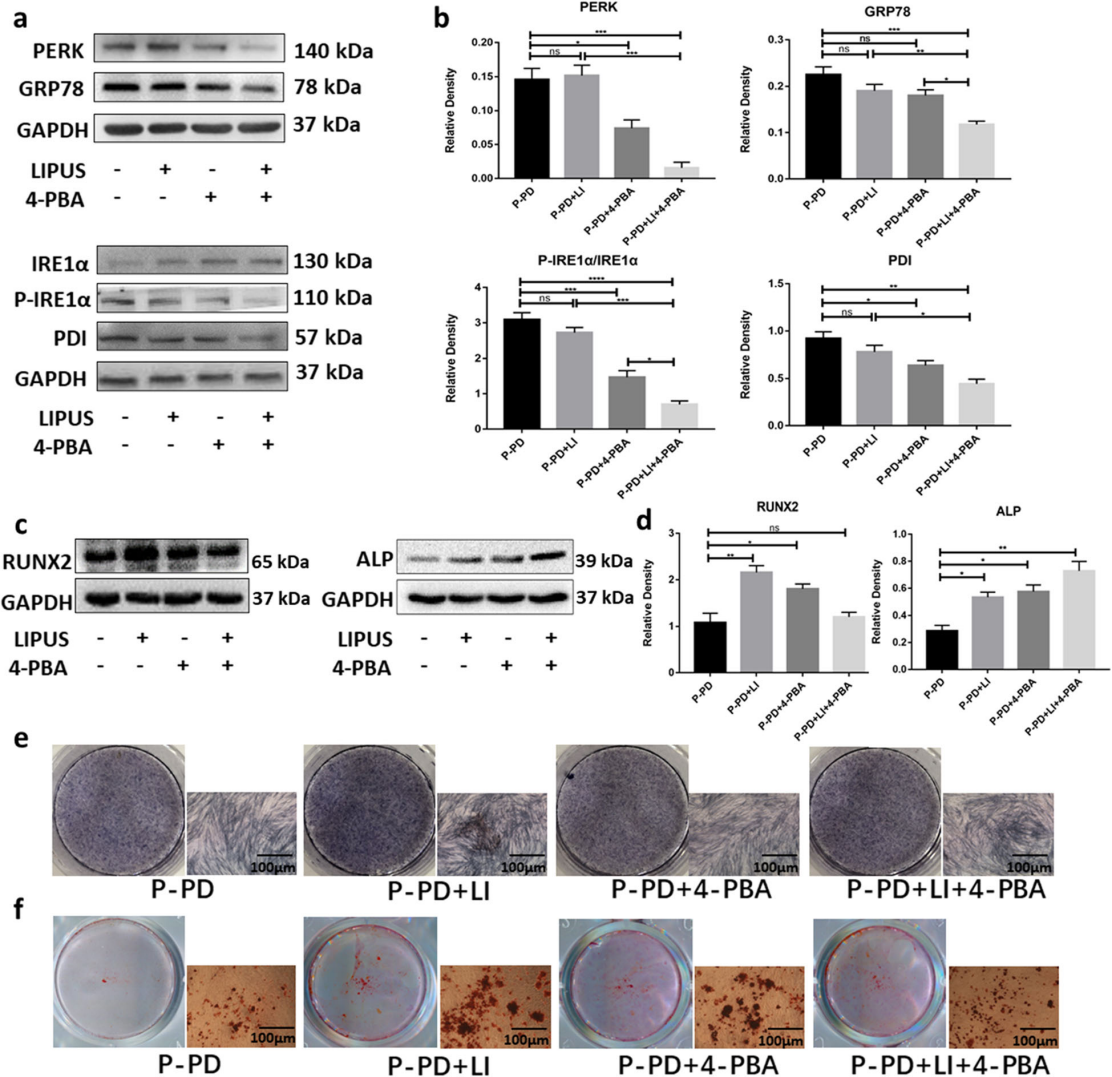
LIPUS affected the UPR-related proteins and enhanced the osteogenesis of P-PD. **a** Western blot analysis of proteins related to ERS including GRP78, PERK, P-IRE1α, and IRE1α. **b** Relative expression levels of the UPR-related proteins (PERK, GRP78, p-IRE1α/IRE1α, and PDI) were measured by ImageJ software (*n* = 3). **c**, **d** Western blot analysis of RUNX2 and ALP (**c**) and the relative density by ImageJ (**d**). **e**, **f** ALP and alizarin red staining were used to detect osteogenic differentiation. Compared to the P-PDLSCs group. The values represent mean ± SD of three independent experiments (ns, no significant; *p < 0 .05; **p < 0 .01; ***p < 0 .001; ****p < 0 .0001). P-PD, P-PDLSCs; H-PD, H-PDLSCs; LI, LIPUS

Then, we used Western blot to detect the UPR-related proteins GRP78, protein kinase receptor-like ER kinase (PERK), IRE1α and phosphorylated IRE1α (p-IRE1α), PDI, and bone-related proteins RUNX2 and ALP. The results showed that UPR-related proteins were reduced in the P-PDLSCs after LIPUS or 4-PBA treatment (Fig. 4a, b), while the expressions of RUNX2 and ALP were significantly increased (Fig. 4c, d). Furthermore, ALP staining showed that LIPUS and/or 4-PBA treatment enhanced the osteogenesis of the P-PDLSCs (Fig. 4e). The degree of matrix mineralization was measured by alizarin red solution after 21 days of induction. There were more mineralized nodules after LIPUS and/or 4-PBA treatment (Fig. 4f), a finding that is consistent with the evidence from ALP staining. These results suggested that the activation of the UPR in P-PDLSCs leads to increased inflammation and reduced osteogenesis. LIPUS can decrease inflammation and induce osteogenic differentiation of P-PDLSCs by reducing the UPR.

LIPUS regulates *Escherichia coli* (*E. coli*) LPS-induced inflammation and improves osteogenesis of H-PDLSCs by reducing the UPR.

**Fig. 5:**
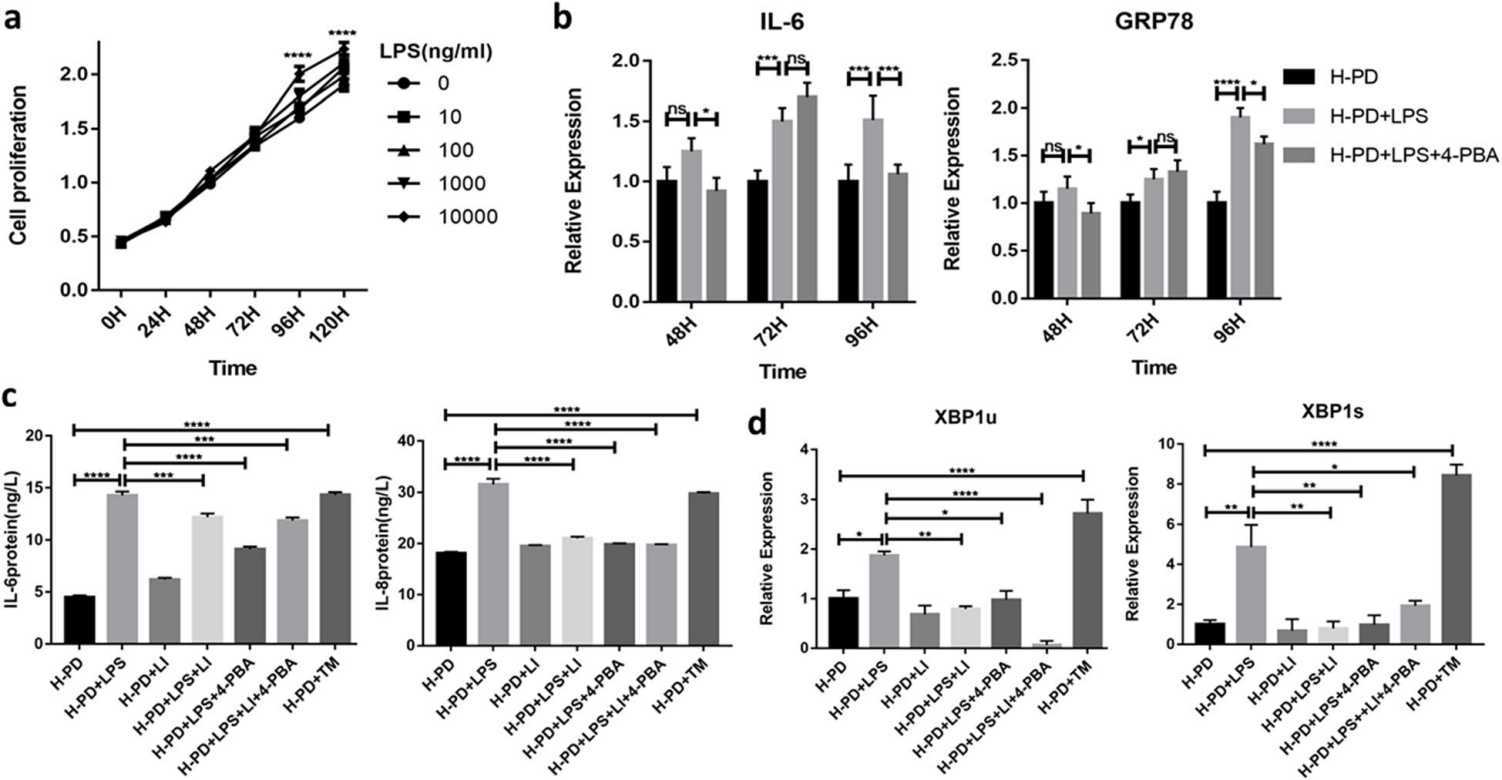
LPS induction simulated inflammatory microenvironment and LIPUS reduced inflammation by reducing UPR. **a** CCK-8 detected the proliferation of H-PDLSCs treated with LPS (0–10,000 ng/ml) and optimized the concentration of LPS (*n* = 5). **b** LPS and 4-PBA treated for different times, and the expression of ER and inflammation-related genes GRP78 and IL-6 were detected by qRT-PCR (*n* = 3). **c** ELISA detected the effects of LIPUS on inflammation-related genes (IL-6, IL-8) of H-PDLSCs induced by LPS (*n* = 3). **d** The expression of ERS-related genes XBP1u and XBP1s by qRT-PCR (*n* = 3). All data are presented as means ± SD. The representative results were from three independent experiments (*p < 0 .05; **p < 0 .01; ***p < 0 .001; ****p < 0 .0001). P-PD, P-PDLSCs; H-PD, H-PDLSCs; LI, LIPUS; H, hour

*E*.*coli* LPS is a commonly used proinflammatory agent that triggers TLR-4 signaling and upregulates the expression of inflammatory factors such as TNF-α, IL-1β, and IL-6 [20, 21]. We used *E. coli* LPS to imitate an inflammatory environment. Cultured H-PDLSCs were treated with different concentrations of LPS (0, 10, 100, 1000, and 10,000 ng/ml) for 5 consecutive days to verify the optimal LPS concentration. We found that 10,000 ng/ml LPS did not affect cell vitality. The cells seemed to proliferate at a higher rate after LPS induction (Fig. 5a). Additionally, to select the optimal time for LPS-induced inflammation and UPR, the expression of IL-6 and GRP78 was measured in cells treated with LPS and/or 4-PBA for different times (48 h, 72 h, and 96 h). The results demonstrated that IL-6 and GRP78 were upregulated after LPS treatment for 72 h and 96 h, while the expression was attenuated by 4-PBA after 96 h of treatment (Fig. 5b). As ERS is a chronic response, we chose to treat samples after stimulation with LPS (10,000 ng/ml) for 96 h.

We used ELISA to examine the effect of 96 h of LPS stimulation on inflammatory factors. After treatment with LIPUS, the ERS inhibitor 4-PBA and agonist tunicamycin (TM) (Solarbio, China) respectively, we found that the expression of IL-6 and IL-8 was significantly upregulated in the LPS and TM groups but was downregulated after treatment with LIPUS and/or 4-PBA (Fig. 5c). Then, we used qRT-PCR to measure the expression of XBP1u and XBP1s, the target genes of IRE1α in the UPR pathway. We found that LPS and TM stimulation resulted in an obvious upregulation of XBP1u and XBP1s, whereas LIPUS and 4-PBA treatment suppressed their expression (Fig. 5d).

**Fig. 6:**
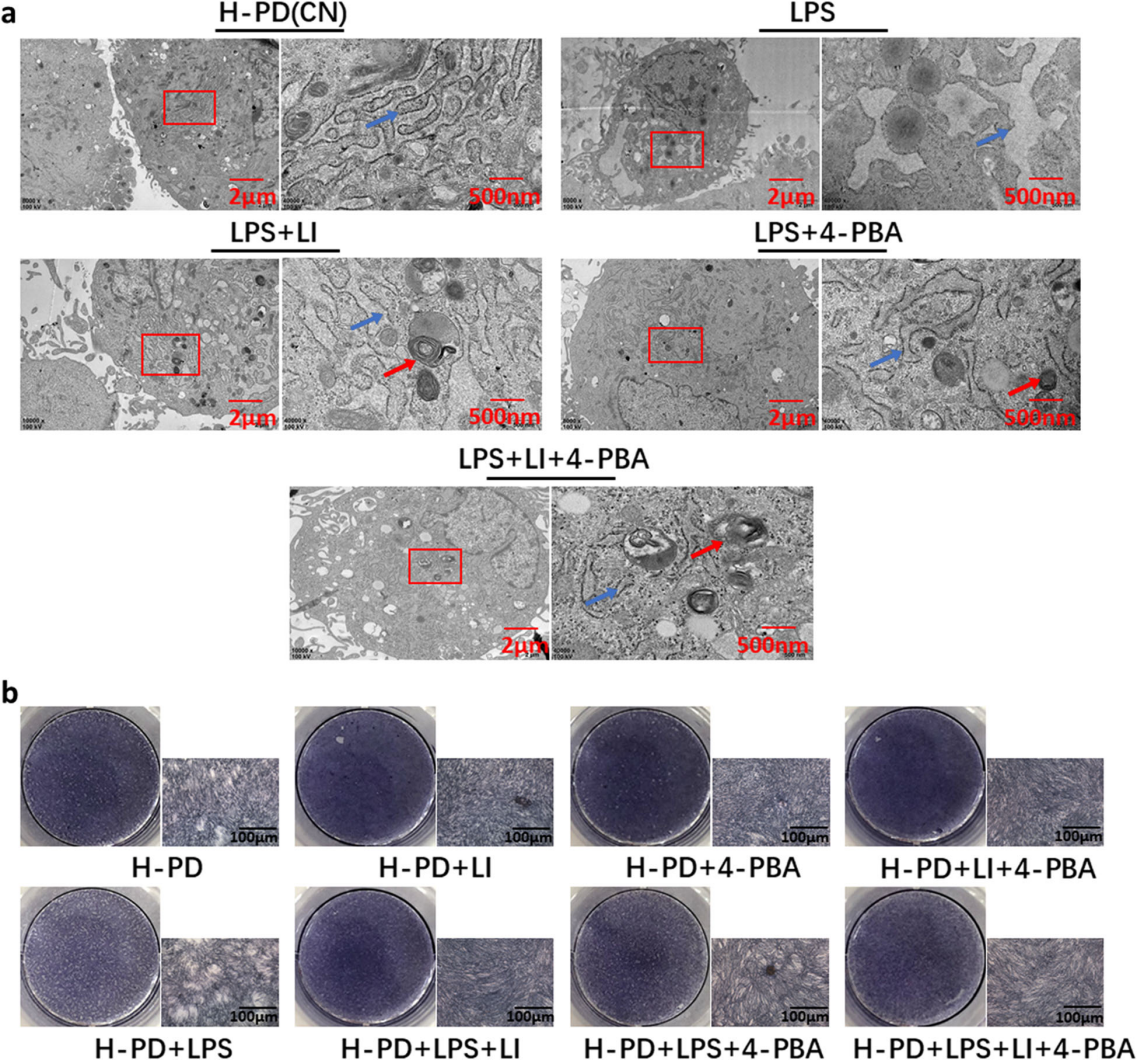
LPS caused ERS and reduced the osteogenic capacity of H-PD. **a** Protective effect of LIPUS on the ultrastructural changes in LPS-induced H-PDLSCs by TEM: LPS (10 μg/ml), 4-PBA (5 μM), and LIPUS (90 mW/cm^2^) were used. Well-arranged rough endoplasmic reticula (RERs) with abundant attached ribosomes were observed in the control group. Rapid proliferation of RERs, some swelled cisternae, and degranulation were shown in cells treated with LPS. Blue arrows indicated ultrastructural changes of ER in H-PDLSCs. Red arrows indicated the autophagosome. **b** ALP staining was used to detect osteogenic differentiation for different groups. The representative results were from three independent experiments. P-PD, P-PDLSCs; H-PD, H-PDLSCs; LI, LIPUS

In addition, we used TEM to observe ultrastructural changes in the ER. Well-formed and well-arranged rough endoplasmic reticula (RERs) were observed in the control group, as was abundant ribosome attachment. After LPS stimulation, the morphology of the ER changed, and some swelled cisternae and degranulation were observed in the cells. LIPUS and/or 4-PBA treatment reversed this change (Fig. 6a). Next, we pretreated cells with or without 4-PBA for 8 h and then treated them with OM containing LPS to observe the effect of continuous LIPUS irradiation for 7 days on the osteogenic differentiation of H-PDLSCs. The results of the ALP staining indicated that LPS highly attenuated osteogenesis contrasted with the control group. However, treatment with LIPUS and/or 4-PBA significantly enhanced osteogenic differentiation. Without LPS treatment, LIPUS and/or 4-PBA alone, the osteogenesis of H-PDLSCs was enhanced (Fig. 6b). These findings indicated that LPS dramatically simulated the inflammatory microenvironment, leading to the upregulation of inflammatory factors in H-PDLSCs and ERS, impairing their osteogenic differentiation ability. However, LIPUS reduced LPS-induced ERS, downregulated the inflammatory response, and enhanced the osteogenesis in the H-PDLSCs.

### In vivo experiments show the effect of LIPUS on experimental periodontitis in rats

**Fig. 7:**
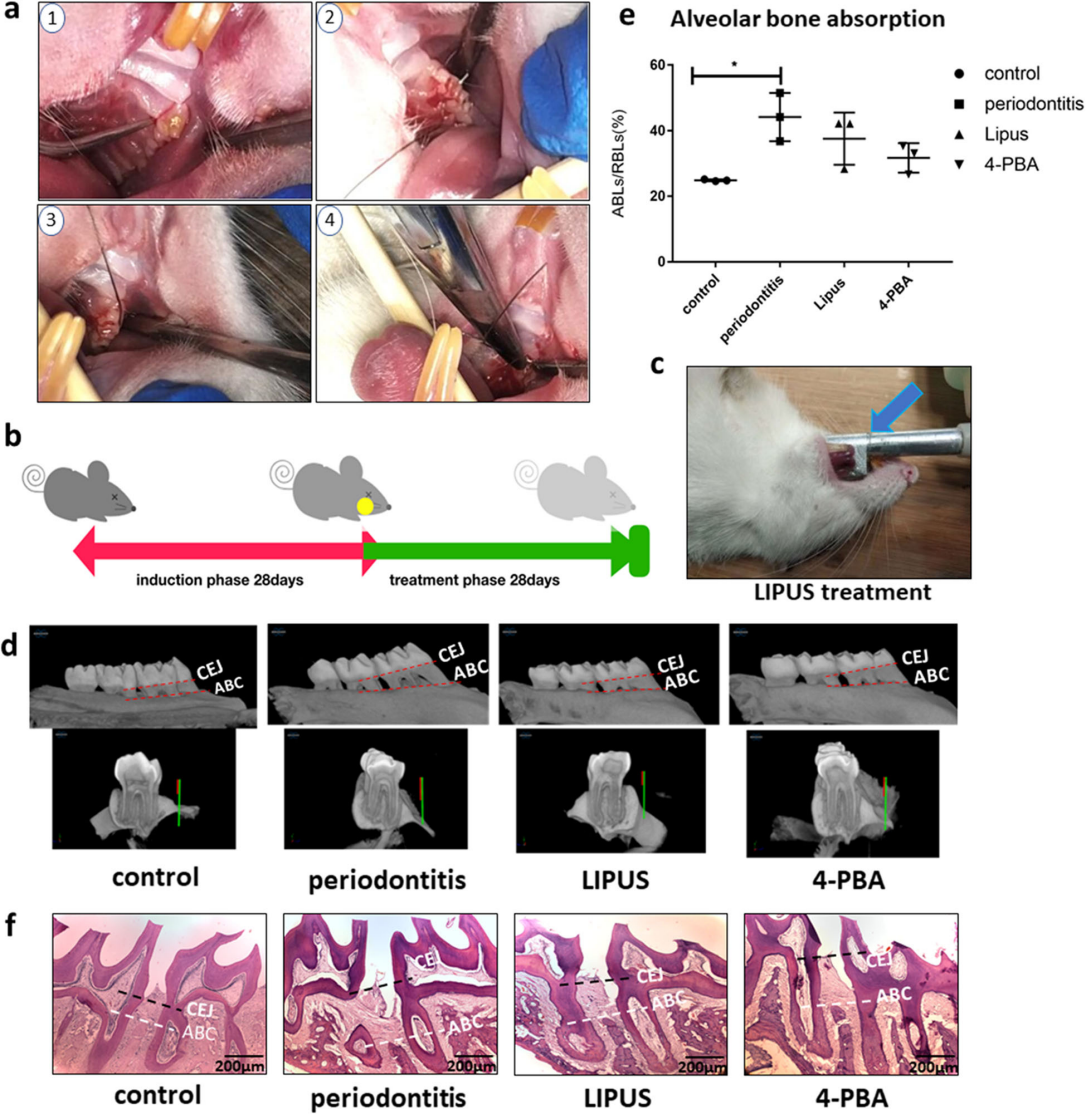
LIPUS and 4-PBA decreased alveolar bone absorption in experimental periodontitis in rats. **a** 4-step surgery procedures including separating the connective gingiva, ringing the ligature wire of the first maxillary molar, fastening the ligature, and ensuring the tight touch to the teeth to induce the experimental periodontitis in rats. **b** Schematic of 2 phases of the experimental periodontitis induction and treatment. **c** Picture of the LIPUS treatment in rat’s oral. The blue arrow is the ultrasonic probe of the LIPUS. **d**, **f** The alveolar bone absorption of SD rats (*n* = 3) was determined by micro-CT (**d**) and H&E staining (**f**). **e** The quantity results of the micro-CT, the absorption of the alveolar bone ratio (ABLs/RBLs) variable is used. The results of micro-CT and H&E staining showed less alveolar bone loss in both the LIPUS and 4-PBA treatment groups compared with the periodontitis group. Guide line CEJ means the parallel line of cemento-enamel junction (CEJ), and guide line ABC means the parallel line of alveolar bone Climax. The representative results were from three independent experiments. The error bars represent the S.D. from the mean values. *p < 0 .05; **p < 0 .01; ***p < 0 .001; ****p < 0 .0001

To determine whether periodontitis triggers the ERS that leads to bone loss and whether the LIPUS and ERS inhibitor rescue bone formation in vivo, we constructed experimental periodontitis models using rats, treating them with LIPUS or the ERS inhibitor 4-PBA. Healthy rats acted as a control group for the assessment of the therapeutic effect. The four groups were as follows: (1) control, (2) experimental periodontitis, (3) LIPUS treatment, and (4) 4-PBA treatment. Each group included five rats. We used ligatures to insert into the distal side and ring the maxillary first molar and injected 0.1 ml LPS (10 μg/ml) four times in total to induce experimental periodontitis (Fig. 7a). The flow of the induction and treatment phases is shown in Fig. 7b. We customized the LIPUS probe for animal experiments so that the probe fit the oral cavity of the rats (Fig. 7c). In addition, the ultrasonic coupling agent was used to conduct LIPUS to the desired treatment area. We found that the LIPUS and 4-PBA treatment groups showed a lower alveolar bone loss ratio (ABLs/RBLs) than did the periodontitis group (Fig. 7d–f). In summary, we found that LIPUS regulates the osteogenic ability of PDLSCs in the inflammatory environment by UPR both in vitro and in vivo.

## Discussion

**Fig. 8:**
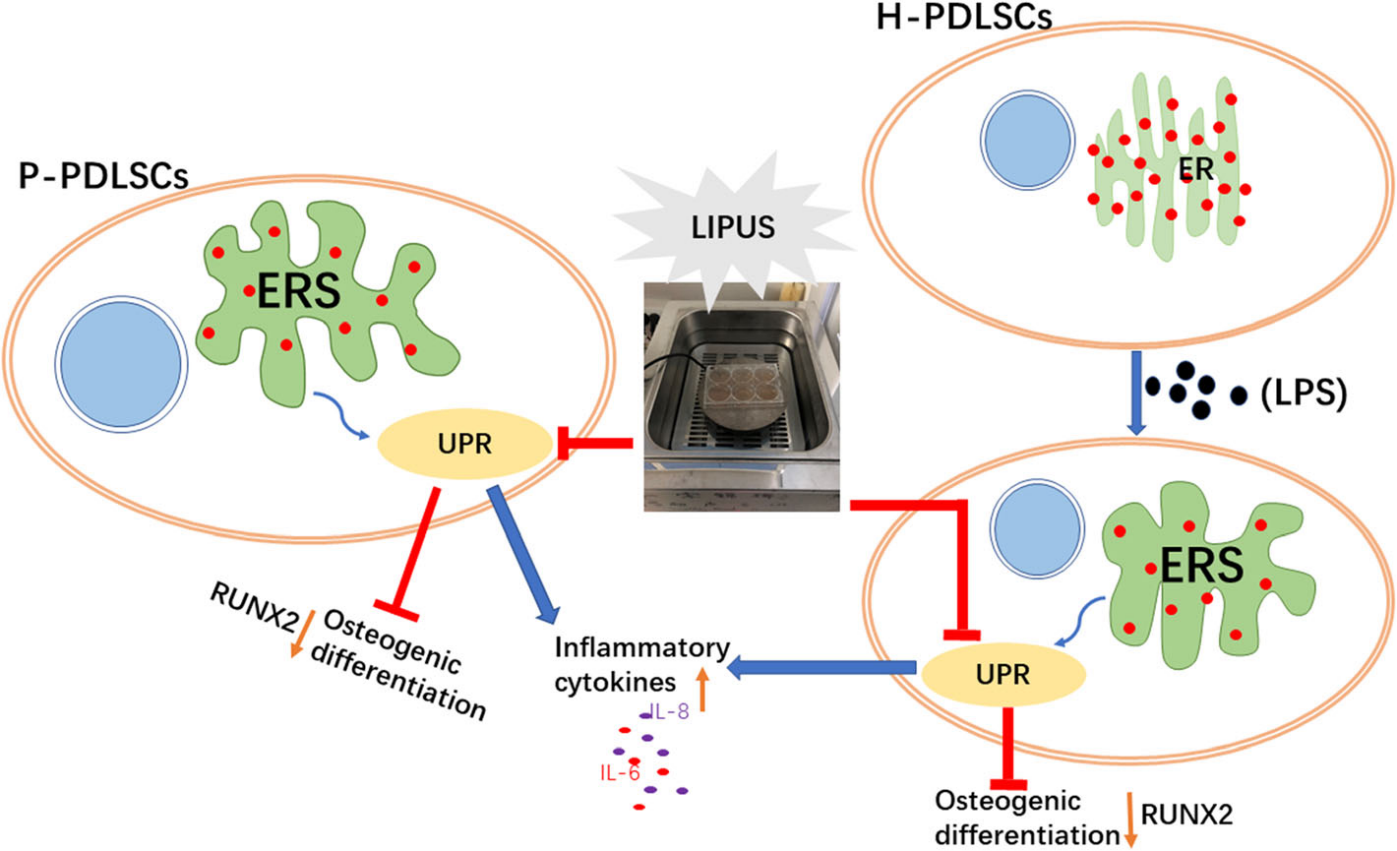
Schematic diagram of this study. The inflammatory environment triggers ERS of PDLSCs, thereby increasing inflammation and decreasing osteogenesis by enhancing the UPR, while LIPUS can inhibit UPR to reduce inflammation and increase osteogenic differentiation

In this study, we explored the effects of LIPUS on the inflammatory response and osteogenic potential of P-PDLSCs derived from inflammatory periodontal tissue and their relationship with ERS. LIPUS downregulated ERS through the UPR, which led to the enhanced osteogenic ability of PDLSCs in the chronic periodontitis environment (Fig. 8). The present study also provided new insights into the application of LIPUS, a non-invasive mechanical stimulation method that played a substantial role in regulating the bone formation capacity of PDLSCs, especially in the chronic inflammatory environment.

Chronic periodontitis causes the excessive proliferation of inflammatory cells, which ultimately damages periodontal connective tissue and causes alveolar bone resorption [22]. Long-term proinflammatory cytokines produced by periodontitis induced prolonged ERS and impaired the osteogenic ability of PDLSCs [11], which made tissue repair difficult in the disrupted area around the tooth. In our current study, by comparing the osteogenic differentiation ability of the H-PDLCs from orthodontics-purposed extracted healthy teeth and the P-PDLSCs from periodontitis patients, we discovered a decrease in the osteogenic capacity of P-PDLSCs as well as an increase in ERS marker expression. One possible explanation for the difference is that increased ERS disrupted the normal function of the endoplasmic reticulum; thus, fewer osteogenic-related proteins can be generated. This process is called the unfolded protein response (UPR), which acts in the endoplasmic reticulum when ERS is triggered [23]. Van Galen et al. reported that, in an inflammatory model, various factors can cause the accumulation of the unfolded proteins leading to ERS [24], and periodontitis is one of these inflammatory diseases. Meanwhile, compared with H-PDLSCs, we found more obvious proliferation of P-PDLSCs according to the CCK-8 assay results, especially after 3 days of culturing. A possible explanation for the increased proliferation rate of P-PDLSCs is that the potential for differentiation was partly impaired by the chronic inflammatory environment, while the proliferation remained normal. These results are consistent with previous studies [25, 26]. We also observed a morphology change in the ER by TEM and found that while the endoplasmic reticulum in the H-PDLCs was well-arranged with ribosomes attached normally, the ER in the P-PDLSCs group was swollen, deformed, and degranulated, which indicated a disruption in the environmental homeostasis of the cell, findings consistent with the LPS-stimulated H-PDLC group. However, after treatment with LIPUS or 4-PBA, the swollen ER was partly relieved. And Juan et al. also observed that strategies for alleviating ERS can reduce the inflammatory response of human umbilical vein endothelial cells induced by LPS [27].

Since healthy PDLSCs are critical for maintaining tissue homeostasis, repair, and remodeling [28], many methods have been used to improve the differentiation ability of PDLSCs. Our previous studies have demonstrated that LIPUS causes increased H-PDLC ALP staining and Runx2 expression and matrix mineralization [16, 29], and it also enhances new bone formation and periodontal regeneration in beagle dog models [30]. However, the effect of LIPUS on P-PDLSCs was unclear, which has limited the application of LIPUS in the treatment of periodontitis, because the inflammatory environment is unavoidable during periodontitis treatment. A study by Cao et al. has shown that suppression of ERS could alleviate chronic inflammation in adipocytes [31]. As a molecular chaperone, 4-PBA reduced excessive ER load caused by mutations or mislocated proteins in the study of liver injury [32] and could inhibit ERS-mediated apoptosis and prevent cerebral ischemia [33]. The ERS inhibitor 4-PBA rescued the osteogenic differentiation of P-PDLSCs in the Xue et al. study [11]. Therefore, we used 4-PBA with which to compare and evaluate the function of LIPUS applied to P-PDLSCs. Interestingly, LIPUS not only showed an effect similar to that of 4-PBA, increasing the formation of mineralized nodules in P-PDLSCs, but also downregulated the expression of the inflammation factors IL-6 and IL-8 at both the mRNA and protein levels. Further study is needed to determine the specific sites of action.

In addition, we mimicked the inflammatory microenvironment in vitro with LPS to observe the effect of LIPUS on ERS, inflammatory expression, and the osteogenesis of H-PDLSCs. LPS is an important virulence factor that is tightly associated with the initiation and development of chronic periodontitis [34, 35]. A previous study reported that LPS was an effective stimulant for inflammatory factor production and bone resorption [36]. Tunicamycin (TM) is a known ERS activator that can cause protein error accumulation in the ER [37, 38]. Our ELISA results suggested that the expression of IL-6 and IL-8 in LPS-treated H-PDLSCs was significantly increased, a finding consistent with the effect of TM. X-box binding protein 1 (XBP-1) is a crucial signaling molecule in the ERS pathways. When ERS is activated, XBP1 is encoded by an unconventional splicing event to generate an effective UPR inducer XBP1s [23, 39]. The expression of XBP1u and XBP1s increased in the LPS-induced H-PDLSCs and decreased after treatment with LIPUS and 4-PBA. The imaging also revealed that both LIPUS and 4-PBA could attenuate the morphology of swollen and degranulated ER. Moreover, ALP and alizarin red staining showed that the osteogenesis was inhibited after LPS induction, while osteogenesis was increased after LIPUS and 4-PBA treatment, and more mineralized calcium nodules were produced. These results suggested that LIPUS downregulates inflammation and promotes osteogenesis in an LPS-induced inflammatory environment by inhibiting ERS. The same results were found in the experimental periodontitis rat models, and the absorption of alveolar bone was rapidly reduced after treatment with 4-PBA or LIPUS.

Our study also highlighted the regulation of UPR when LIPUS and 4-PBA were used to treat the PDLSCs. The UPR-related proteins PERK and PDI were downregulated after LIPUS and 4-PBA treatment. As a result, the decreased phosphorylation of IRE1α drove the reduction in the downstream transcription factor XBP-1. The rescue of osteogenic differentiation can likely be attributed to the activation of the self-protection activity of the PDLSCs because in the TEM images, we could see autophagosomes present in the cells after treatment. Autophagy is commonly thought to be a cytoprotective response to UPR [40] with decreased UPR leading to re-established cell hemostasis. Furthermore, the UPR has three pathways from stress to homeostatic regulation [41]. Our current study did not reveal sufficient evidence to uncover the specific pathway included in the use of LIPUS, so further study is needed.

We also discovered that the use of LIPUS and 4-PBA could reverse of alveolar bone absorption in the experimental periodontitis rat model. We employed the ligatures to the maxillary first molar and injected LPS, which is a widely used method of inducing periodontitis in animals [42]. The bone loss ratio (ABLs/RBLs) of the treatment groups was sharply decreased compared with the periodontitis group. The ratio is more objective to show bone absorption than the distance between alveolar bone climax (ABC) and enamel cementum junction (CEJ), as shown in the figures. Because the molar root shape varies a lot in each rat involved in the experiment and it could influence the variable of bone loss distance measured in micro-CT [43].

In conclusion, we demonstrated that P-PDLSCs derived from periodontitis were in the ERS state, resulting in increased inflammation and decreased osteogenic ability compared to H-PDLCs. LIPUS alleviated ERS and inflammation while improving the bone formation ability of P-PDLSCs in vivo and in vitro. In future research, we will explore the specific regulation of the UPR pathway involved upon the application of LIPUS and the related mechanism(s).

## Conclusion

We conducted a preliminary study about the effect of LIPUS on PDLSCs in an inflammatory state and the relationship with ERS. The results suggest that LIPUS enhances the osteogenic ability and reduces the inflammation of P-PDLSCs through the UPR. This study points to an underlying mechanism of applying LIPUS and provides new possibilities for periodontal tissue regeneration.

## Data Availability

The datasets used and analyzed during the current study are available from the corresponding author on reasonable request.

## Abbreviations

PDLSCs: Periodontal ligament stem cells
P-PDLSCs: Periodontal ligament stem cells derived from patients with periodontitis
H-PDLSCs: Periodontal ligament stem cells derived from healthy teeth
LIPUS: Low-intensity pulsed ultrasound
OM: Osteogenic medium
ERS: Endoplasmic reticulum stress
UPR: Unfolded protein response
*P*. *gingivalis*: *Porphyromonas gingivalis*
PDL: Periodontal ligament
CTE: Cell tissue engineering
MSCs: Mesenchymal stem cells
LPS: Lipopolysaccharides
PE: Phycoerythrin
ALP: Alkaline phosphatase
CCK-8: Cell Counting Kit-8
TEM: Transmission electron microscopy
qRT-PCR: Quantitative real-time PCR
IL-6: Interleukin-6
IL-8: Interleukin-8
GRP78: Glucose-regulated protein78
IRE1α: Inositol requiring enzyme1α
ATF4: Activating transcription factor
CHOP: Transcription factor C/EBP homologous protein
XBP1: X-box binding protein 1
GAPDH: Glyceraldehyde 3-phosphate dehydrogenase
RUNX2: Runt-related transcription factor 2
ELISA: Enzyme-linked immunosorbent assay
SD: Sprague-Dawley
HE: Hematoxylin-eosin
4-PBA: 4-Phenyl butyric acid
*E*. *coli*: *Escherichia coli*
RERs: Rough endoplasmic reticula
TM: Tunicamycin

## References

[1] Hajishengallis G (2015). Periodontitis: from microbial immune subversion to systemic inflammation. Nat Rev Immunol.

[2] Wei W, Xiao X, Li J (2019). Activation of the STAT1 pathway accelerates periodontitis in mice. J Dent Res.

[3] Smiley CJ, Tracy SL, Abt E (2015). Evidence-based clinical practice guideline on the nonsurgical treatment of chronic periodontitis by means of scaling and root planing with or without adjuncts. J Am Dental Assoc (1939).

[4] Gaudilliere DK, Culos A, Djebali K (2019). Systemic immunologic consequences of chronic periodontitis. J Dent Res.

[5] Sahni V, Tibrewal S, Bissell L, Khan W (2014). The role of tissue engineering in Achilles tendon repair: a review. Curr Stem Cell Res Therapy.

[6] Khan WS, Malik A (2012). Stem cell therapy and tissue engineering applications for cartilage regeneration. Curr Stem Cell Res Ther.

[7] Seo BM, Miura M, Gronthos S, Bartold PM, Batouli S, Brahim J (2004). Investigation of multipotent postnatal stem cells from human periodontal ligament. Lancet..

[8] Wang L, Shen H, Zheng W, Tang L, Yang Z, Gao Y (2011). Characterization of stem cells from alveolar periodontal ligament. Tissue Eng Part A.

[9] Chen SC, Marino V, Gronthos S, Bartold PM (2006). Location of putative stem cells in human periodontal ligament. J Periodontal Res.

[10] Liu N, Shi S, Deng M (2011). High levels of β-catenin signaling reduce osteogenic differentiation of stem cells in inflammatory microenvironments through inhibition of the noncanonical Wnt pathway. J Bone Mineral Res.

[11] Xue P, Li B, An Y, Sun J, He X, Hou R, et al. Decreased MORF leads to prolonged endoplasmic reticulum stress in periodontitis-associated chronic inflammation. Cell Death Differentiation. 2016;23(11). 10.1038/cdd.2016.74. 10.1038/cdd.2016.74 PMC5071575 27447113

[12] Cao SS, Zimmermann EM, Chuang BM (2013). The unfolded protein response and chemical chaperones reduce protein misfolding and colitis in mice. Gastroenterology..

[13] Domon H, Takahashi N, Honda T, Nakajima T, Tabeta K, Abiko Y (2009). Up-regulation of the endoplasmic reticulum stress-response in periodontal disease. Clin Chim Acta.

[14] Yamada H, Nakajima T, Domon H, Honda T, Yamazaki K (2015). Endoplasmic reticulum stress response and bone loss in experimental periodontitis in mice. J Periodontal Res.

[15] Yang SY, Wei FL, Hu LH, Wang CL (2016). PERK-eIF2α-ATF4 pathway mediated by endoplasmic reticulum stress response is involved in osteodifferentiation of human periodontal ligament cells under cyclic mechanical force. Cell Signal.

[16] Hu B, Zhang Y, Zhou J (2014). Low-intensity pulsed ultrasound stimulation facilitates osteogenic differentiation of human periodontal ligament cells. PLoS One.

[17] Zhang X, Hu B, Sun J, Li J, Liu S, Song J (2017). Inhibitory effect of low-intensity pulsed ultrasound on the expression of lipopolysaccharide-induced inflammatory factors in U937 cells. J Ultrasound Med.

[18] Su Z, Xu T, Wang Y (2019). Low-intensity pulsed ultrasound promotes apoptosis and inhibits angiogenesis via p38 signaling-mediated endoplasmic reticulum stress in human endothelial cells. Mol Med Rep.

[19] Kim HJ, Jeong JS, Kim SR, Park SY, Chae HJ, Lee YC (2013). Inhibition of endoplasmic reticulum stress alleviates lipopolysaccharide-induced lung inflammation through modulation of NF-κB/HIF-1α signaling pathway. Sci Rep.

[20] Olmos-Ortiz A, Déciga-García M, Preciado-Martínez E (2019). Prolactin decreases LPS-induced inflammatory cytokines by inhibiting TLR-4/NFκB signaling in the human placenta. Mol Hum Reprod.

[21] Nogueira-Filho G, Rosa BT, Santos PF (2014). Whole-blood cultures from patients with chronic periodontitis respond differently to Porphyromonas gingivalis but not Escherichia coli lipopolysaccharide. J Periodontol.

[22] Yang H, Aprecio RM, Zhou X (2014). Therapeutic effect of TSG-6 engineered iPSC-derived MSCs on experimental periodontitis in rats: a pilot study. PLoS One.

[23] Calfon M, Zeng H, Urano F (2002). IRE1 couples endoplasmic reticulum load to secretory capacity by processing the XBP-1 mRNA. Nature..

[24] Peter VG, Antonija K, Nathan M, Kent DG, Timothy F, Chambers JE (2014). The unfolded protein response governs integrity of the haematopoietic stem-cell pool during stress. Nature..

[25] Tang HN, Xia Y, Yu Y, Wu RX, Gao LN, Chen FM (2016). Stem cells derived from “inflamed” and healthy periodontal ligament tissues and their sheet functionalities: a patient-matched comparison. J Clin Periodontol.

[26] Um S, Lee JH, Seo BM (2018). TGF-β2 downregulates osteogenesis under inflammatory conditions in dental follicle stem cells. Int J Oral Science.

[27] Chen J, Zhang M, Zhu M, Gu J, Song J, Cui L, et al. Paeoniflorin prevents endoplasmic reticulum stress-associated inflammation in lipopolysaccharide-stimulated human umbilical vein endothelial cells via the IRE1α/NF-κB signaling pathway. Food Function. 2018;9(4).DOI: 10.1039/C7FO01406F. 10.1039/c7fo01406f 29594285

[28] Konstantonis D, Papadopoulou A, Makou M, Eliades T, Basdra E, Kletsas D (2014). The role of cellular senescence on the cyclic stretching-mediated activation of MAPK and ALP expression and activity in human periodontal ligament fibroblasts. Exp Gerontol.

[29] Chen D, Xiang M, Gong Y (2019). LIPUS promotes FOXO1 accumulation by downregulating miR-182 to enhance osteogenic differentiation in hPDLCs. Biochimie..

[30] Wang Y, Qiu Y, Li J, Zhao C, Song J (2018). Low-intensity pulsed ultrasound promotes alveolar bone regeneration in a periodontal injury model. Ultrasonics..

[31] Cao W, Zhang T, Feng R (2019). Hoxa5 alleviates obesity-induced chronic inflammation by reducing ER stress and promoting M2 macrophage polarization in mouse adipose tissue. J Cell Mol Med.

[32] Perlmutter DH (2002). Chemical chaperones: a pharmacological strategy for disorders of protein folding and trafficking. Pediatr Res.

[33] Qi X, Hosoi T, Okuma Y, Kaneko M, Nomura Y (2004). Sodium 4-phenylbutyrate protects against cerebral ischemic injury. Mol Pharmacol.

[34] Seo T, Cha S, Kim TI, Lee JS, Woo KM (2012). Porphyromonas gingivalis-derived lipopolysaccharide-mediated activation of MAPK signaling regulates inflammatory response and differentiation in human periodontal ligament fibroblasts. J Microbiol (Seoul, Korea).

[35] Kato H, Taguchi Y, Tominaga K, Umeda M, Tanaka A (2014). Porphyromonas gingivalis LPS inhibits osteoblastic differentiation and promotes pro-inflammatory cytokine production in human periodontal ligament stem cells. Arch Oral Biol.

[36] Kim DS, Shin MR, Kim YS (2015). Anti-inflammatory effects of glutamine on LPS-stimulated human dental pulp cells correlate with activation of MKP-1 and attenuation of the MAPK and NF-κB pathways. Int Endod J.

[37] Dorner AJ, Wasley LC, Raney P, Haugejorden S, Green M, Kaufman RJ (1990). The stress response in Chinese hamster ovary cells. Regulation of ERp72 and protein disulfide isomerase expression and secretion. J Biol Chem.

[38] Takatsuki A, Arima K, Tamura G (1971). Tunicamycin, a new antibiotic. I. Isolation and characterization of tunicamycin. J Antibiot (Tokyo).

[39] Kaser A, Lee AH, Franke A (2008). XBP1 links ER stress to intestinal inflammation and confers genetic risk for human inflammatory bowel disease. Cell..

[40] Lee WS, Yoo WH, Chae HJ (2015). ER stress and autophagy. Curr Mol Med.

[41] Walter P, Ron D (2011). The unfolded protein response: from stress pathway to homeostatic regulation. Science..

[42] Bhattarai G, Poudel SB, Kook SH, Lee JC (2016). Resveratrol prevents alveolar bone loss in an experimental rat model of periodontitis. Acta Biomater.

[43] Ho KN, Lee SY, Huang HM (2017). Damping ratio analysis of tooth stability under various simulated degrees of vertical alveolar bone loss and different root types. Biomed Eng Online.

